# Phylogenomic and Macroevolutionary Evidence for an Explosive Radiation of a Plant Genus in the Miocene

**DOI:** 10.1093/sysbio/syab068

**Published:** 2021-08-16

**Authors:** Hanghui Kong, Fabien L Condamine, Lihua Yang, A J Harris, Chao Feng, Fang Wen, Ming Kang

**Affiliations:** Key Laboratory of Plant Resources Conservation and Sustainable Utilization, South China Botanical Garden, Chinese Academy of Sciences, Guangzhou 510650, China; Institut des Sciences de l’Evolution de Montpellier (Université de Montpellier ∣ *CNRS* ∣ IRD ∣ EPHE), Place Eugène Bataillon, Montpellier 34095, France; Key Laboratory of Plant Resources Conservation and Sustainable Utilization, South China Botanical Garden, Chinese Academy of Sciences, Guangzhou 510650, China; Key Laboratory of Plant Resources Conservation and Sustainable Utilization, South China Botanical Garden, Chinese Academy of Sciences, Guangzhou 510650, China; Key Laboratory of Plant Resources Conservation and Sustainable Utilization, South China Botanical Garden, Chinese Academy of Sciences, Guangzhou 510650, China; Guangxi Institute of Botany, Guangxi Zhuang Autonomous Region and the Chinese Academy of Sciences, Guilin 541006, China; Key Laboratory of Plant Resources Conservation and Sustainable Utilization, South China Botanical Garden, Chinese Academy of Sciences, Guangzhou 510650, China; Center of Conservation Biology, Core Botanical Gardens, Chinese Academy of Sciences, Guangzhou 510650, China

## Abstract

Mountain systems harbor a substantial fraction of global biodiversity and, thus, provide excellent opportunities to study rapid diversification and to understand the historical processes underlying the assembly of biodiversity hotspots. The rich biodiversity in mountains is widely regarded as having arisen under the influence of geological and climatic processes as well as the complex interactions among them. However, the relative contribution of geology and climate in driving species radiation is seldom explored. Here, we studied the evolutionary radiation of *Oreocharis* (Gesneriaceae), which has diversified extensively throughout East Asia, especially within the Hengduan Mountains (HDM), using transcriptomic data and a time calibrated phylogeny for 88% (111/126) of all species of the genus. In particular, we applied phylogenetic reconstructions to evaluate the extent of incomplete lineage sorting accompanying the early and rapid radiation in the genus. We then fit macroevolutionary models to explore its spatial and diversification dynamics in *Oreocharis* and applied explicit birth–death models to investigate the effects of past environmental changes on its diversification. Evidence from 574 orthologous loci suggest that *Oreocharis* underwent an impressive early burst of speciation starting ca. 12 Ma in the Miocene, followed by a drastic decline in speciation toward the present. Although we found no evidence for a shift in diversification rate across the phylogeny of *Oreocharis*, we showed a difference in diversification dynamics between the HDM and non-HDM lineages, with higher diversification rates in the HDM. The diversification dynamic of *Oreocharis* is most likely positively associated with temperature-dependent speciation and dependency on the Asian monsoons. We suggest that the warm and humid climate of the mid-Miocene was probably the primary driver of the rapid diversification in *Oreocharis*, while mountain building of the HDM might have indirectly affected species diversification of the HDM lineage. This study highlights the importance of past climatic changes, combined with mountain building, in creating strong environmental heterogeneity and driving diversification of mountain plants, and suggests that the biodiversity in the HDM cannot directly be attributed to mountain uplift, contrary to many recent speculations.[East Asian monsoons; environmental heterogeneity; Hengduan Mountains; incomplete lineage sorting; *Oreocharis*; past climate change; rapid diversification; transcriptome.]

Intense proliferations of taxa in a short time, or explosive radiations, have intrigued evolutionary biologists for more than a century (Osborn 1902; Givnish 2015; Lagomarsino et al. 2016; Simões et al. 2016; Cai et al. 2020). Rapid radiations are common phenomena in nature, with numerous phylogenetic studies documenting extreme cases of explosive radiations for many taxa such as, in animals (e.g., López-Fernández et al. 2010; Townsend et al. 2011; Cai et al. 2020; Roycroft et al. 2020), plants (e.g., Valente et al. 2010; Lagomarsino et al. 2016; Carlsen et al. 2018; Pouchon et al. 2018; Testo et al. 2019), and even microbes (Morlon et al. 2012). Evolutionary biologists have long endeavored to determine what underlying factors govern the evolutionary dynamics of these explosive radiations, which are believed to have often occurred early in the history of a lineage (Benton 2009; Moen and Morlon 2014; Condamine et al. 2019). Broadly, most hypotheses attribute either biotic factors or abiotic factors to early rapid radiations. Specifically, biotic factors such as life history traits, hybridization, introgression, and species interactions may have conferred high potential for species to diversify into new niches, until the niche become saturated (López-Fernández et al. 2010; Rabosky and Glor 2010). Alternatively, abiotic factors, namely geological events and climate change, could favor radiation such as during warm periods (Condamine et al. 2019) or episodes of mountain building (Lagomarsino et al. 2016; Esquerré et al. 2019).

Notably, mountain ranges are often associated with rapid radiations as well as high biodiversity (Drummond et al. 2012; Favre et al. 2015; Lagomarsino et al. 2016; Kong et al. 2017; Antonelli et al. 2018; Condamine et al. 2018; Esquerré et al. 2019; Muellner-Riehl 2019). Taken together, this seems to suggest that mountain building drives rapid radiations, possibly by establishing topographic heterogeneity and creating novel habitats where species evolve and diversify (Antonelli et al. 2018; Rahbek et al. 2019). However, regarding mountain building as the sole driver in diversification of mountain species is likely an oversimplification (Hoorn et al. 2013; Renner 2016; Condamine et al. 2018). For example, several studies have suggested the importance of past global climate changes in plant radiations (Janssens et al. 2009; Mao et al. 2010; Madriñán et al. 2013; Favre et al. 2016). These different processes are, however, not likely to be independent of one another, and may act simultaneously or in succession on a taxonomic group. Therefore, disentangling different historical processes, such as mountain building, from others and quantifying the relative contribution of each is key to elucidating the assembly and maintenance of the diversity of mountain species.

In southwestern China, the Hengduan Mountains (HDM) comprises a global biodiversity hotspot located along the southeastern Qinghai-Tibet Plateau (QTP) and is a major contributor to the overall high regional levels of species richness and endemism. The HDM exhibits dramatic variation in climate and topography and, perhaps consequently, supports one of the most endemic-rich temperate floras in the world, with an estimated 12,800 species accounting for ca. 43% of the total number of Chinese seed plants in an area of about 500,000 km$^{2}$ with ca. 5.2% of area in China (Sun et al. 2017). The accumulation of this extreme diversity is due in large part to a large number of species-rich rapid radiations, especially involving at least 16 genera with over 100 extant species (Sun et al. 2017). Thus, the HDM represents an excellent system for studying the drivers of evolutionary radiation in plants.

Uplift of the HDM has often been implicated in shifts of diversification rates in various plant groups over the last million years (e.g., Favre et al. 2015, 2016; Ebersbach et al. 2017; Ye et al. 2019). However, recent fossil and tectonic evidence support that the HDM began its rise in the early Eocene and reached its current elevation by the early Oligocene (Su et al. 2018; Spicer et al. 2020, 2021; Xiong et al. 2020), which largely predates previous estimates that pointed to a Miocene (Hoke et al. 2014; Li et al. 2015) or even Pliocene (Favre et al. 2015) uplift. Moreover, mountain building in eastern Asia triggered climatic change, especially in the intensity and patterns of the regional monsoons (Farnsworth et al. 2019). Specifically, the overall elevation of the region achieved during the Oligocene blocked dry continental air and yielded great intensification of the East Asian monsoons during the Miocene (Farnsworth et al. 2019). This intensification of the monsoon appears to coincide with rapid radiation in several plant lineages outside of other global climatic changes during the Miocene, such as the Miocene climatic optimum, and outside of specific episodes of mountain building (Ding et al. 2020). Thus, in light of the latest fossil and geological evidence on the uplift history of the HDM, it is necessary to reevaluate the relative contributions of mountain building, East Asian monsoons, and global climate change in driving rapid radiations within the region, and generating species diversity and endemism in Southwest China.

The species-rich genus *Oreocharis* (Gesneriaceae), which has very high morphological diversity (Möller et al. 2011), is a relevant model system to disentangle the possible drivers of a rapid radiation. The monophyletic genus *Oreocharis* comprises ca. 120 species that are predominantly distributed in China, with ca. 10 species also occurring in northern Indo-China (Möller et al. 2011, 2016). Within *Oreocharis*, species exhibit strongly divergent floral characters such as in corolla shape and coloration, fertility of stamens, anther shape and dehiscence mode morphology (Fig. 1a), but they show little variation in vegetative habit and fruit structure (Möller et al. 2011). *Oreocharis* likely underwent an early rapid radiation based on previous molecular phylogenetic analyses with using *trnL-F* and ITS sequences, which revealed short branches at deep nodes (Möller et al. 2011). Furthermore, recent work has shown a higher diversification rate of *Oreocharis* than the background diversification rate for the other gesneriad lineages in China (Roalson and Roberts 2016). The species are lithophytic (i.e., grow on rock or stony soil) and/or terrestrial and mainly comprise rosette herbs that occupy subtropical, humid, and monsoonal forests at elevations of 50–2,500 m, but sometimes even occurring as high as 3,000 m within the HDM region. Few are known from the tropical rainforests of Hainan and southern Yunnan Provinces (Supplementary Appendix S1 available on Dryad at http://dx.doi.org/10.5061dryad.9cnp5hqjf). The genus has three centers of diversity: one lies within the HDM and its adjacent areas in Southwest China (average altitude ca. 4,000 m), another is in the Wuling Mountains in Central China (average altitude ca. 1,000 m), and additionally within the Nanling Mountains in South China (average altitude ca. 1,000 m) (Fig. 1b). This distribution facilitates comparisons of diversification between different mountain systems. Based on the current understanding of the evolutionary history and distribution of biodiversity of *Oreocharis*, the genus constitutes an excellent model system to examine the relative contributions of mountain building, the monsoon, and past climate changes as possible drivers of its rapid radiation.

**Figure 1.**
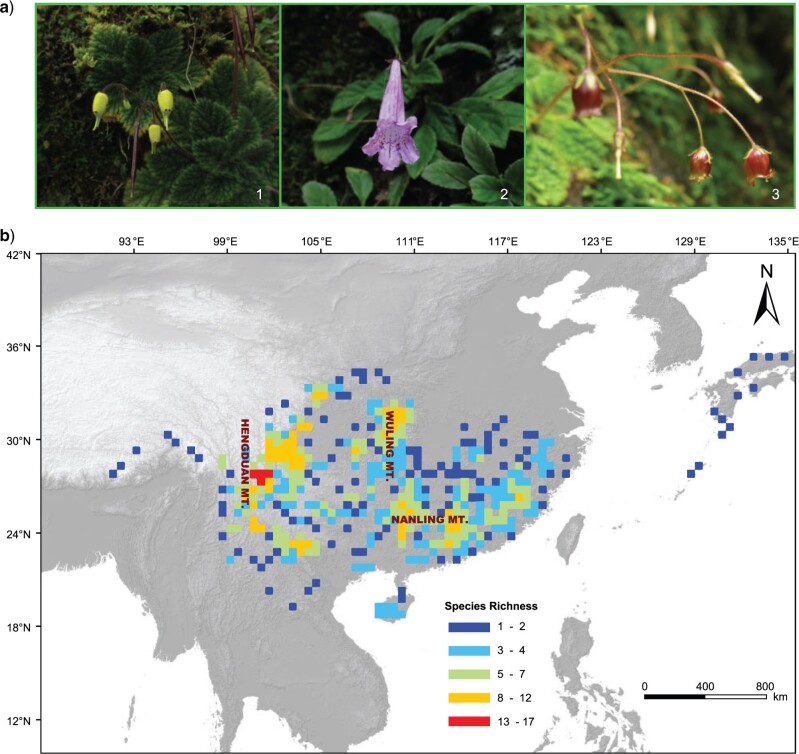
Morphology and species richness pattern of *Oreocharis*. a) Representative species of *Oreocharis*, showing their strongly divergent floral characters, especially corolla shape and coloration. 1. *Oreocharis urceolata* (K.Y. Pan) Mich. Möller & A. Weber; 2. $O.$  *wentsaii* (Z.Y. Li) Mich. Möller & A. Weber; 3. $O.$  *parviflora* L. Cai and Z.K. Wu (© Lihua Yang). b) The geographic distribution of species richness of *Oreocharis*. The species distribution data were obtained from our extensive field investigations from 2016 to 2019 and from locality records of digitized specimen data (http://www.cvh.ac.cn/). The species richness pattern was calculated with equal-area square grid cells of 0.5° using Biodiverse (version 2.1) (Laffan et al. 2010).

In this study, we used transcriptomic data to reconstruct the evolutionary history of the species-rich genus *Oreocharis*. We used the transcriptomes to provide a species-level molecular phylogeny of the genus based on sampling 111 of the total 126 species of *Oreocharis* and evaluate the extent of incomplete lineage sorting (ILS) accompanying the rapid radiation. We then fit macroevolutionary models to investigate the spatial and diversification dynamics and compare the diversification rates among lineages from different mountain systems. We also applied diversification models to infer the effect of global temperature changes and fluctuations in the strength of East Asian monsoons during the evolutionary history of *Oreocharis*.

## Materials and Methods

### Taxon Sampling, RNA Extractions, and Illumina Sequencing

We collected samples throughout the entire geographic range of *Oreocharis* in China and adjacent regions (except Japan) during our field investigations from 2016 to 2019 (Supplementary Appendix S1 and S2 available on Dryad). These samples represented 111 species (123 populations, including varieties) out of 120 described *Oreocharis* species, as well as six undescribed species based on morphological characters (labeled as *Oreocharis* sp. nov. 1–6) (Supplementary Appendix S2 available on Dryad). In the field, we recorded geographic coordinates using a Garmin-eTrex GPS device. From each population, we randomly collected whole living plants, enclosed the roots and lower portions of the petioles in damp moss, which we found in the field and bound with twine, in order to prevent desiccation. We brought the collected plants to the greenhouse at South China Botanical Garden, Chinese Academy of Sciences for rearing in cultivation. Using these cultivated samples, we collected young, fresh leaves, which were frozen immediately in liquid nitrogen before storage at $-$80° C. Based on the latest phylogenies of *Oreocharis* (Möller et al. 2011; Roalson and Roberts 2016), we selected outgroups comprising five closely related genera, *Aeschynanthus buxifolius*, *Aeschynanthus moningeriae*, *Anna mollifolia*, *Cyrtandra hawaiensis*, *Didymocarpus cortusifolius*, and *Petrocodon dealbatus*, plus two more distantly related outgroups, *Henckelia tibetica* and *Microchirita hamosa*. However, these two distant outgroups were absent during the identification of orthologs according to the rooted ingroups method (for obtaining single-copy genes, described below). For all field collections, we obtained voucher specimens, which we deposited in the herbarium of South China Botanical Garden, Chinese Academy of Sciences (IBSC). All information regarding the samples obtained for this study is available in Supplementary Appendix S2 available on Dryad.

From each individual, we extracted total RNA from the frozen leaves using an RNeasy Plant Mini Kit (Qiagen, Valencia, CA, USA), which included treatment with RNase-free DNase I (TaKaRa, Dalian, China) to eliminate DNA contamination. We determined the quality and quantity of the extracted RNA of each sample using the agarose gel electrophoresis and a 2100 Bioanalyzer (Agilent Technologies).We sent the purified RNA to Novogene Corporation (Tianjin, China) for preparation of cDNA libraries and sequencing on an Illumina NovaSeq 6000 platform to generate 150 bp paired-end (PE) reads. We deposited all newly obtained, raw reads in the NCBI Sequence Read Archive under Bioproject accession number PRJNA649046 and BioSample accession numbers from SAMN15655841 to SAMN15655969 (Supplementary Appendix S2 available on Dryad).

### Reads Filtering, Orthologs Identification, and Phylogenetic Analysis

We performed quality control on the raw reads to ensure the reliability of downstream analyses. Specifically, we filtered raw reads using a custom Perl script (QC_pe.pl, https://github.com/scbgfengchao/; Feng et al. 2017) to remove the low-quality reads (i.e., reads containing unknown/ambiguous base higher than 5%, more than 20% bases with quality scores less than 20) to obtain clean reads. We then assembled the cleaned reads *de novo* for each individual sample using Trinity 2.6.6 (Grabherr et al. 2011) with K-mer size of 32 and under default settings for other parameters. We used the longest isoform from each Trinity subcomponent to generate unigenes using our script (Trinity2Unigene.pl, https://github.com/scbgfengchao/; Feng et al. 2020), and we reduced redundant unigenes using CD-HIT-EST 4.7 (with the parameter -c 0.98) (Fu et al. 2012). We identified coding regions using TransDecoder (Haas et al. 2013) by performing BLAST search against known, annotated proteins in the UniProt and Protein Family (Pfam) databases with an E-value cutoff of 1.0E$-$5.

We used OrthoFinder 2.3.3 (Emms and Kelly 2015, 2019) to classify the orthogroups of proteins from all the species of *Oreocharis* and outgroups (with the parameter -S diamond -og). We excluded the orthogroups with the following criteria (Ran et al. 2018): 1) sequences are absent in at least one species; 2) the mean copy number of an orthogroup is more than five; and 3) the median copy number of an orthogroup is more than two. We identified homologs and orthologs from all the samples based on the approach of Yang and Smith (2014) (https://bitbucket.org/yangya/phylogenomic_dataset_construction, accessed on October 15, 2019). After excluding orthogroups based on the criteria above, we aligned the remaining orthogroups using MAFFT 6.864b (Katoh and Standley 2013), inferred two rounds maximum likelihood phylogenies using RAxML 8.2.11 (Stamatakis 2014), and pruned terminal branches that were longer than 0.2 and more than 10 times longer than its sister. We also pruned branches longer than 0.5. According to definition of Yang and Smith (2014) and Yang et al. (2015), the remaining trees comprised potential homologs. Using these trees, we obtained 1:1 orthologs (i.e., the orthologs represented in single copy for each species) by pruning the trees of homologs using the rooted ingroups method (i.e., prune by extracting ingroup clades and then cut paralogs from root to tip) (Yang and Smith 2014), allowing for missing species (i.e., the pruned trees could contain missing taxa for outgroups or ingroups). To minimize the impact of the phylogenetic inference error, we excluded orthologous loci from the analyses if they were absent in all species of the outgroup or did not yield a monophyletic clade for the outgroup species in the gene tree reconstructions. For the remaining orthologs, we generated a total of four data sets allowing 5%, 25%, 50%, and 75% of taxa to be missing at each locus.

For each of the four data sets, we performed phylogenomic analyses using maximum likelihood (ML) with a supermatrix implemented in IQ-TREE 2.0.3 (Minh et al. 2020), a sited-based coalescent method with SVDquartets (Chifman and Kubatko 2014), and a multispecies summary coalescent model with ASTRAL-III 5.6.3 (Zhang et al. 2018a). For the ML analysis, we identified the best-fit substitution model for each locus via ModelFinder (Kalyaanamoorthy et al. 2017) implemented in IQ-TREE, and determined node support with 1,000 ultrafast bootstrap (UFBS) replicates (Hoang et al. 2018). UFBS values were considered strong support when they were higher or equal to 95%. We used the SVDquartets method as implemented in PAUP* 4.0a167 (Swofford 2003) to infer species trees. This method utilizes all site patterns from sequence data directly to evaluate the three possible splits on a four-taxon tree and, thus, may avoid some of the errors from gene tree estimation. We performed 1,000 bootstrap replicates for each data set, and set the number of randomly sampled quartets to 500,000 for assessing support.

For the coalescent-based analysis, we first performed phylogenetic inference with IQ-TREE with substitution model selection and tree inference separately for each gene and calculated nodal support with 1,000 UFBS replicates. To avoid arbitrary topologies detrimentally influencing the species tree, branches arising from nodes with less than 10% UFBS support value were collapsed on each tree (Zhang et al. 2017) using the “nw_ed” function of Newick utilities (Junier and Zdobnov 2010). We input the resulting gene tree files with collapsed nodes into ASTRAL with the “-t 3” option to calculate the species tree with local posterior probability support (Sayyari and Mirarab 2016).

We measured the differences among trees from different gene data sets based on the average Robinson–Foulds (RF) distance (Robinson and Foulds 1981) with the “-rf_all” function in IQ-TREE. We performed measurements for trees resulting from the supermatrix approach, SVDquartets method, and ASTRAL approach independently. We used a custom R script (https://figshare.com/articles/software/compare_toplogy/13712920) to visualize the discordance among three species trees from different approaches.

### Incongruence between Gene Trees and Species Trees

To assess the degree of discordance between our gene and species trees, we applied a multidimensional scaling (MDS) method to plot pairwise RF distances between all gene tree and species tree topologies (Duchêne et al. 2018). MDS provides a visualization of the variance among gene tree topologies and their distance from the consensus species tree topology using different dimensions. To visualize the relationship between information content of each gene and per-gene tree distance from the species tree, we colored MDS points by average bootstrap support values.

### Quartet Sampling

The quartet sampling (QS) method is a quartet-based evaluation system that offers an alternative measure for branch support (Pease et al. 2018). Under the QS method, one taxon is randomly resampled from each of the four subsets at the focal branch, and then the likelihood values of all three possible phylogenies based on the aligned sequence data are evaluated. This method returns the quartet concordance (QC), quartet differential (QD), quartet informativeness (QI), and quartet fidelity (QF) to assess the confidence, consistency, informativeness of the internal nodes, and the reliability of each terminal branch (Pease et al. 2018). We conducted the QS analysis using quartet_sampling.py (https://www.github.com/fephyfofum/quartetsampling, accessed on May 10, 2020) with a maximum of 200 quartet replicates. We evaluated the likelihood scores of the possible topologies for each quartet sample using RAxML 8.2.11 (Stamatakis 2014) on the whole data set. The log-likelihood threshold cutoff was set as default (–lnlike 2), and the minimum sites to be sampled for all taxa in a given quartet was set to 20,000.

### Evaluating incomplete lineage sorting

Incomplete lineage sorting (ILS) is one of the most fundamental causes of discordance among gene trees. Among new, innovative algorithms for addressing discordance is *MSCquartets*, which is designed for inference of species trees and species networks under the multispecies coalescent (MSC) model of ILS as well as species tree hypothesis-testing (Allman et al. 2021; Rhodes et al. 2021). Specifically, *MSCquartets* determined the number of the three possible quartet topologies for each set of four taxa among all gene trees. These are referred to as quartet count concordance factor (qcCF) and can be compared to generate $P$-values for judging the fit of gene trees to a species tree. All quartet concordance factors for all choices of four taxa can be viewed using a simplex plot to give a comprehensive view of discordance across the entire gene tree data set (Allman et al. 2021).

To test the degree of ILS in *Oreocharis*, we carried out *MSCquartets* analyses. We used the function quartetTreeTest with “T1 model” implemented in the *MSCquartets* package under different rejection levels with $\alpha =$ 0.01, 0.001, 1e$-$04, and 1e$-$06. For each result, we generated a simplex plot for all four-taxon subsets with color-coded symbols that show either that particular qcCFs are consistent with the MSC of ILS on a species tree at the specified level of statistical significance or that some mechanism other than ILS most likely explains discordance (e.g., substantial gene tree inference error, hybridization, or gene duplication and loss). When ILS is present, symbols may lie in closer proximity to the centroid indicating substantial ILS or closer to the vertex indicating little ILS.

### Estimation of Divergence Times

There are presently no known fossils of the Gesneriaceae (Roalson and Roberts 2016; no occurrence in Paleobiology Database, accessed on March 10, 2020). Therefore, we used secondary calibrations based on Roalson and Roberts (2016) to calibrate a molecular clock and divergence times of lineages within *Oreocharis*. Roalson and Roberts (2016) used 12 external fossil calibration points and geologic ages to estimate divergence times among lineages represented by 768 species of Gesneriaceae and found that the crown age of *Oreocharis* was 12.33 Ma with a 95% highest posterior density (HPD) between 7.92 and 16.60 Ma. We applied the 95% HPD of age estimates from that study as secondary calibration points using uniform prior distributions and used the concatenated data set with 574 genes for the dating analyses.

To estimate divergence times within *Oreocharis*, we carried out molecular dating analyses with BEAST 2.6.1 (Bouckaert et al. 2019) under an uncorrelated lognormal clock model with a uniform prior bounded between 0 and 1. We set the DNA substitution models to the GTR$+\Gamma$ model and the birth–death model as the branching process prior (Condamine et al. 2015) both with default prior parameters. We performed two independent runs (with different random starting seeds) in BEAST comprising 50 million generations with sampling every 5,000 generations of the Markov Chain Monte Carlo (MCMC). We used Tracer 1.7.1 (Rambaut et al. 2018) to assess convergence and to check that the effective sample sizes (ESS) of the parameters estimated in each run were greater than 200, indicating stationarity. We discarded the first 25% of trees as burn-in and generated the maximum clade credibility (MCC) chronogram from the remaining trees with mean node ages and 95% HPD with TreeAnnotator of the BEAST package.

### Temporal Dynamic of Diversification

We carried out several different diversification analyses to test the temporal dynamic of diversification. These comprised lineages-through-time (LTT) in the R-package *APE* 5.3 (Paradis and Schliep 2019), BAMM 2.5.0 (Rabosky et al. 2013) to estimate speciation and extinction rates through time and across clade, *RPANDA* 1.2 (Morlon et al. 2016) to cross-validate results from BAMM, and the compound Poisson process on Mass-Extinction Times (CoMET, May et al. 2016) model as implemented in the R-package *TESS* 2.1 (Höhna et al. 2016) to test for abrupt changes in diversification rates.

To visualize the temporal dynamic of diversification in* Oreocharis*, we used the LTT function in *APE* 5.3 to plot 7,500 trees (after 25% burn-in) from the dating analysis, as well as the MCC tree. We tested the hypothesis that diversification rates of *Oreocharis* were best represented by an early, rapid radiation followed by a decline of diversification, and we applied different models to infer the suitability of this hypothesis. For all these models, we set a global sampling fraction for *Oreocharis* of 0.88 (111 sampled over 126 species, including the six undescribed species), and other parameters were set to default values.

We used BAMM to assess whether diversification rates remained constant or varied exponentially during the evolutionary history, detect the frequency of diversification rate shifts, and where these shifts occurred. The BAMM analyses comprised two MCMC runs of 50 million generations with sampling every 5,000 generations. We used the R-package *BAMMtools* 2.5.0 (Rabosky et al. 2014) to process the output of the MCMC, analyze the posterior distribution of rates allowing the estimation of the diversification rates through time and the best configuration of the diversification rate shifts. We discarded the first 25% of the sampled data as burn-in.

We used the R-package *RPANDA* to cross-validate the BAMM inferences because there are debates on the reliability of rate estimates produced by BAMM (Moore et al. 2016; Meyer and Wiens 2017, but see Rabosky et al. 2017; Rabosky 2018). Within *RPANDA*, we performed time-dependent diversification models as implemented by Morlon et al. (2016) with two constant-rate and four time-dependent models to detect whether rates varied through time and how they varied as follows: 1) speciation is constant and extinction is null (Bcst); 2) both speciation and extinction are constant (BcstDcst); 3) speciation varies through time and extinction is null (BTimeVar); 4) speciation varies through time and extinction is constant (BTimeVarDcst); 5) speciation is constant and extinction varies through time (BcstDTimeVar); and 6) speciation and extinction are both varying through time (BTimeVarDTimeVar). We compared all models according to the corrected Akaike information criterion (AICc), the $\Delta$AIC and the Akaike weight (AIC$\omega )$. To test whether specificities of the environmental curve matter for the statistical support, we smoothed the temperature curve with df $=$ 2 in the spline interpolation (Clavel and Morlon 2017; Condamine et al. 2019), which retains the declining trend but removes accurate features of the curve. We then compared AIC values from the smoothed temperature curve to the original values and assessed if the best model was still supported (i.e., no false positives for the model selection).

We applied CoMET to jointly estimate time-continuous rates and the impact of abrupt changes in diversification or the presence of punctual extinction events. In CoMET, we ran the MCMC for 10 million generations with default priors and allowed for mass extinctions.

### Testing Drivers of Diversification

We tested the hypotheses that changes in diversification rates might be due to environmental factors, such as climatic shifts. Specifically, within the distribution region of the genus, we speculated that paleotemperatures and East Asian monsoons might have had consequences for the diversification of *Oreocharis* as inferred for other gesneriad (Kong et al. 2017). To test this hypothesis, we used a model implemented in *RPANDA* that allows speciation and extinction rates to vary according to an environmental variable, such as temperature or monsoons, that changes through time (Condamine et al. 2013, 2019). We designed four paleoenvironment-dependent models to detect a relationship between the changes of diversification rates and environment as follows 1) speciation rate is varying with environment and extinction rate is null (BEnvVar); 2) speciation rate is varying with environment and extinction rate is constant (BEnvVarDcst); 3) speciation rate is constant and extinction rate is varying with environment (BcstDEnvVar); and 4) speciation and extinction rates are both varying with environment (BEnvVarDEnvVar). Here, environment refers to either past temperatures or East Asian monsoons. We derived the temperature data from the well-known Cenozoic temperature curves published and updated by Zachos et al. (2001, 2008) and obtained the East Asian monsoons data according to the hematite/goethite proxy, which is measured by 565 and 435 nm wavelengths in the color spectra of the freshly cut core from the Ocean Drilling Program (ODP) Site 1148 in the South China Sea (Clift et al. 2014).

The change in diversification can also be interpreted as the effect of competition for resources or niche availability (e.g., Phillimore and Price 2008). Thus, we investigated the hypothesis that an early rapid radiation (whether lineages diversified rapidly in their early stages) was followed by slower rates of speciation as niches were filled and became saturated, reaching their ecological limits (Rabosky 2009, 2013). To accomplish this, we used the diversity-dependent diversification as implemented in the R-package* DDD* 4.2 (Etienne and Haegeman 2012) to infer the effect of diversity on speciation and extinction rates. Within *DDD*, we used the function “*dd_ML*” to fit five models 1) speciation declines linearly with diversity and no extinction (DDL), 2) speciation declines linearly with diversity and extinction (DDL$+$E), 3) speciation declines exponentially with diversity and extinction (DDX$+$E), 4) extinction increases linearly with diversity (DD$+$EL), and 5) extinction increases exponentially with diversity (DD$+$EX). The initial carrying capacity was set to the current species diversity, and the carrying capacity (K) is estimated according to the models and parameters.

We tested whether distribution in the HDM influenced diversification rates using Binary State Speciation and Extinction (BiSSE) and Hidden State Speciation and Extinction (HiSSE) models in the R-packages *diversitree* 0.9-7 (FitzJohn 2012) and* hisse* 1.9.6 (Beaulieu and Meara 2016), respectively. We applied the HiSSE model to incorporate unmeasured factors (i.e., hidden states) that could impact diversification rates besides the focal trait, which was distribution in the HDM or not, coded as 1 and 0, respectively. We allowed unlinked rates of speciation ($\lambda_{0}$, $\lambda_{1})$, extinction ($\mu_{0}$, $\mu_{1})$, and transitions (q$_{01}$, q$_{10})$ associated with the two states for the trait. For the HiSSE model, we set two hidden states (A, B) contained within each observed states (i.e., states 0A, 0B, 1A, 1B), speciation and extinction rates varying independently across all four states, and transition rates between all observed and hidden states free to vary except for dual transitions (e.g., q0A to q1B, q1A to q0B). We optimized the fit of all aforementioned models by maximum likelihood and evaluated model performance based on AICc. To test whether our diversification results were biased due to type I error (Rabosky and Goldberg 2015), we estimated the $\Delta$AIC between the best model and a null model (i.e., with no state dependence), and compared this $\Delta$AIC to a distribution of $\Delta$AIC as estimated from 1,000 simulated trait data and analyzed with the two same BiSSE models. Finally, we assessed the effect of different root states on the diversification rates between HDM and non-HDM lineages by performing additional BiSSE analyses with the root state set to either 0 or 1 (Goldberg and Igić 2008).

### Historical Biogeography

To reconstruct the ancestral area of origin of *Oreocharis*, we inferred ancestral ranges and dispersal events across the phylogeny. Based on vegetation types and the species composition, we defined nine geographic regions representing the sampled distribution of *Oreocharis* according to the floristic regions of China proposed by Huang et al. (2016): A, Hengduan Mountains Region; B, Yunnan Plateau Region; C, Yunnan, Myanmar and Thailand Border Region; D, Central China Region; E, Yunnan, Guizhou and Guangxi Region; F, Beibu Gulf Region; G, East China Region; H, Lingnan Mountains Region; and I, South Sea Region. The presence/absence for each species in each region is shown in Supplementary Appendix S2 available on Dryad. We performed ancestral range estimation on the MCC tree (without outgroups, Harris et al. 2013), using the Dispersal-Extinction-Cladogenesis (DEC, Ree and Smith 2008) model of range evolution as implemented in RASP 20200510 (Yu et al. 2020). We used the DEC model without founder-event dispersal parameter (Matzke 2014), which appears to have both conceptual and statistical flaws (Condamine et al. 2018; Ree and Sanmartín 2018). The maximum areas at each node were set to two based on the largest range of any extant species (Supplementary Appendix S2 available on Dryad).

## Results

### Transcriptome Data

For each transcriptome, we generated between 15,814,973 and 34,476,222 filtered, clean paired-end reads with Q30 values higher than 90% representing 123 populations of *Oreocharis*. These paired-end reads were assembled into 116,833 (*O. obtusidentata*) to 327,758 (*O. acutiloba*) transcripts per transcriptome. The length of N50 for the transcripts ranged from 369 (*O. acutiloba*) to 1,798 bp (*O. pinnatilobata*), and the total number of genes with matches in Pfam ranged from 21,616 (*O. shweliensis*) to 48,143 (*O. acutiloba*) with an average length of 861 bp. Additional sequence information is summarized in Supplementary Appendix S2 available on Dryad.

### Orthologs Filter and Phylogenetic Analyses

#### Filtering of orthologs based on missing data

When we allowed 75% missing taxa for each gene, we obtained 2,360 orthologous genes. After filtering orthologous genes that were not represented among the outgroup species or did not result in the outgroup species forming a clade in phylogenetic reconstruction, we obtained 1027 orthologous genes. For the filtered data sets of 5%, 25%, and 50% missing taxa for each gene, we obtained 33, 240, and 574 orthologous genes, respectively, and the final actual amounts of missing data (base pairs) for entire supermatrix ranged from 3.5% to 40.8% (Supplementary Appendix S3a available on Dryad). Final alignments for all four data sets are available in Supplementary Appendix S4 available on Dryad.

#### Conflicts among species trees and gene trees

The average and pairwise RF distances among species trees are presented in Supplementary Appendix S3a available on Dryad. The ASTRAL tree inferred from the 574 gene trees with up to 50% missing taxa for each gene showed the least conflict among the 12 species trees, with the lowest average distance, 51.2%, to other trees. Therefore, we used this tree for downstream analyses. The variance of RF distance among inferred species trees was much lower than that among inferred gene trees. No tree based on any one of the 574 genes was completely consistent with the overall species tree topology as inferred from the concatenated and coalescent analyses based on the data sets with varying levels of missing taxa for each gene (Supplementary Appendix S3b available on Dryad). Individual gene-tree topologies estimated with IQ-TREE received average UFBS values ranging from 38.57% to 94.63%. The distances for genes in tree space from the consensus species trees were not clearly correlated with the average UFBS, that is, the genes with lower average UFBS were not always further away from the species trees than those with higher average UFBS.

#### Phylogeny of Oreocharis

**Figure 2.**
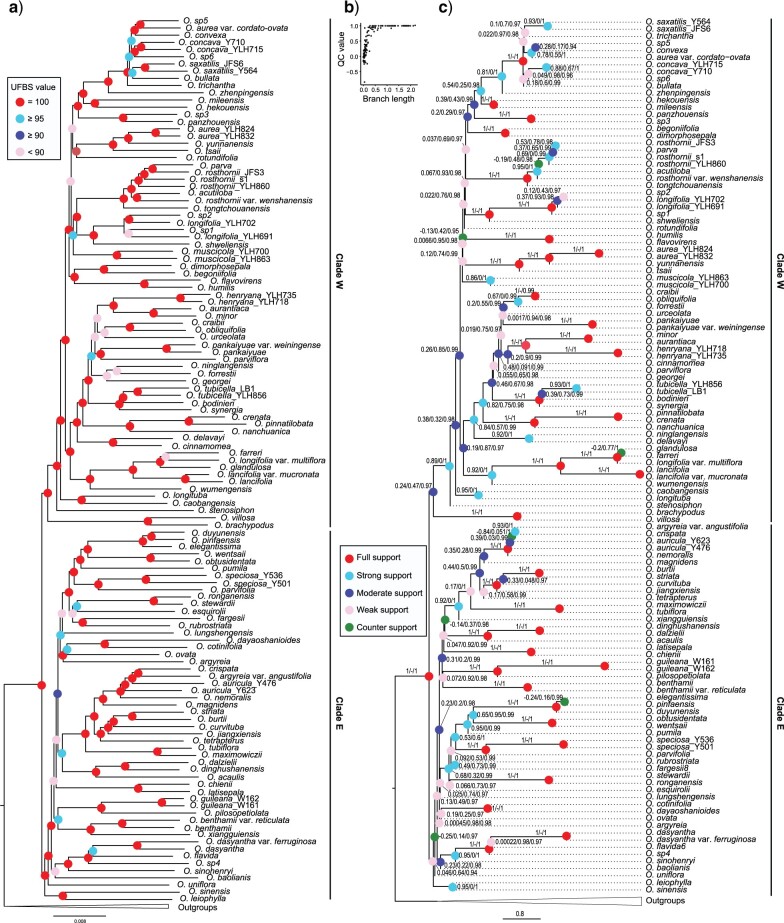
Phylogeny of *Oreocharis* with IQ-TREE and ASTRAL. a) A maximum likelihood (ML) phylogeny of *Oreocharis* from IQ-TREE based on the concatenated dataset with 574 loci and up to 50% missing taxa for each locus. Bootstrap support based on 1,000 ultrafast bootstrap replicates for each node were indicated with colored cycle, where bright red represents UFBS $=$ 100, or full support; turquoise represents 100 >UFBS $\ge$ 95, or strong support; blue represents 95>UFBS$\ge$90, or moderate support; pink represents UFBS < 90, or weak support. b) Strong positive relationship between Quartet Concordance (QC) scores and branch length. c) QC/Quartet Differential (QD)/Quartet Informativeness (QI) scores for internal branches of the phylogeny of *Oreocharis* using multispecies summary coalescent model with ASTRAL. In the heat map coloration of branches bright red represents 1/-/1, or full support; turquoise represents 0.5/0.1 to 0.98/0.97, or strong support; pink represents 0.2/0.2 to 0.99/0.97, or moderate support; purple represents 0.05/0.1 to 0.96/0.97, or weak support; and green represents $-$0.2/0.1 to 0.77/0.95, or counter support, according to Pease et al. (2018).

Node support was evaluated with either UFBS, local posterior probability (LPP), or bootstrap support (BS). The results showed maximal node support for *Oreocharis* in all phylogenomic analyses (Fig. 2; Supplementary Appendices S5–S7 available on Dryad). Two main clades of *Oreocharis*, here after called Clade West (W) and Clade East (E), were robustly recovered by all different methods of inference (Fig. 2; Supplementary Appendices S5–S7 available on Dryad). These two main clades are roughly separated by the Yungui Plateau (Supplementary Appendix S10 available on Dryad). Several taxa had variable phylogenetic positions under different methods of inference, but all phylogenetic variance was within the two major clades, not between them (Supplementary Appendix S10 available on Dryad). There was a strong positive relationship between internal branch lengths and nodal support based on LPP (Supplementary Appendices S7 and S9 available on Dryad), and the conflicting nodes were always estimated with relative low node support, suggesting that conflicts occurred most often at short internodes.

We chose the tree inferred with 574 genes data set and ASTRAL as the reference tree to discuss phylogenetic relationships within *Oreocharis* (Supplementary Appendix S7 available on Dryad). The topology showed a grade of *O. leiophylla* $+$  *O. sinensis* and *O. uniflora* as subtending to all other *Oreocharis* in Clade E. Most species within Clade E were distributed in Central to East China, except *O. parvifolia* and *O. fargesii*, both of which occurred in the adjacent region of Southwestern China (Supplementary Appendix S1 available on Dryad). All the species within Clade W were distributed in the mountains of Southwest China. *Oreocharis longituba*, *O. caobangensis*, *O. stenosiphon*, *O. villosa,* and *O. brachypodus* formed a basal grade within Clade W, while other species of *Oreocharis* in the clade formed two monophyletic groups, which contained all sampled species from the Hengduan Mountains.

### Quartet Sampling Analyses

Quartets were largely informative at all nodes (mean QI of 0.99). The mean quartet concordance (QC) for the branches was 0.55. We found 8.7% of branches had QC values less than $-$0.05 (i.e., counter-support for an alternative quartet topology). Similar to the relationship between node supports and internal branch lengths, there was a strong positive relationship between branch lengths and QC (Fig. 2b). Full support (QC $=$ 1.0) was more often recovered in relatively recent clades than in ancient clades. Well-supported nodes always had long internal branches, and lower QC values were always associated with short branch lengths, possibly due to the rapid radiation and ILS occurring in the early-diverging lineages (Fig. 2c). The mean quartet fidelity (QF) for all species of the ingroup was 0.76, with the lowest QF value recovered for *O. argyreia* (0.46), but according to our quartet sampling (QS) analyses (Pease et al. 2018), there is no evidence for “rogue taxa” in the current phylogeny of *Oreocharis*.

### The Extent of ILS

**Figure 3.**
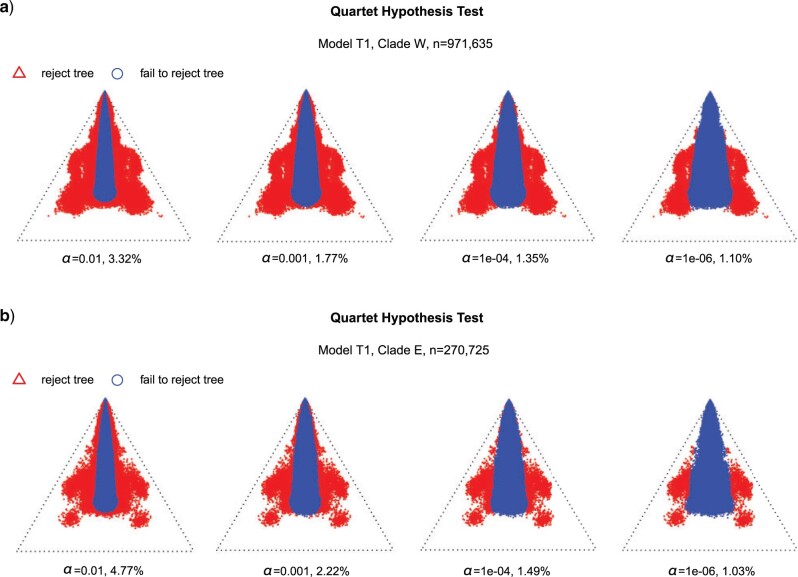
Simplex plots of quartet concordance factors (qcCFs) under the multispecies coalescent (MSC) model of ILS with T1 model based on the species tree inferred with ASTRAL. The analyses were performed under different values of $\alpha$ for rejection levels (from 1e$-$06 to 0.01). Red triangles in the plot indicate rejection of the MSC on the species tree, blue circles indicate probable ILS. a) The simplex plot of qcCFs for Clade W with exhaustive quartet number of 971,635. b) The simplex plot of qcCFs for Clade E with exhaustive quartet number of 270,725.

There were 71 taxa within Clade W and 52 taxa within Clade E in the analyses of *MSCquartets*. The exhaustive quartet number was 971,635 for Clade W and 270,725 for Clade E, respectively. Despite different values of $\alpha$ for rejection levels (from 1e$-$06 to 0.01), the fractions of the rejected trees were relatively stable under the T1 model (from 1.10% to 3.32% for Clade W, and from 1.03% to 4.77% for Clade E; Fig. 3), suggesting that the quartet trees of most species agree with the ASTRAL topology under the MSC model. The simplex plots showed most blue circles lay in closer proximity to the centroid, which indicating that there was substantial ILS among the gene trees and was probably very common within *Oreocharis*.

### Estimation of Divergence Times and Diversification

#### Dating

**Figure 4.**
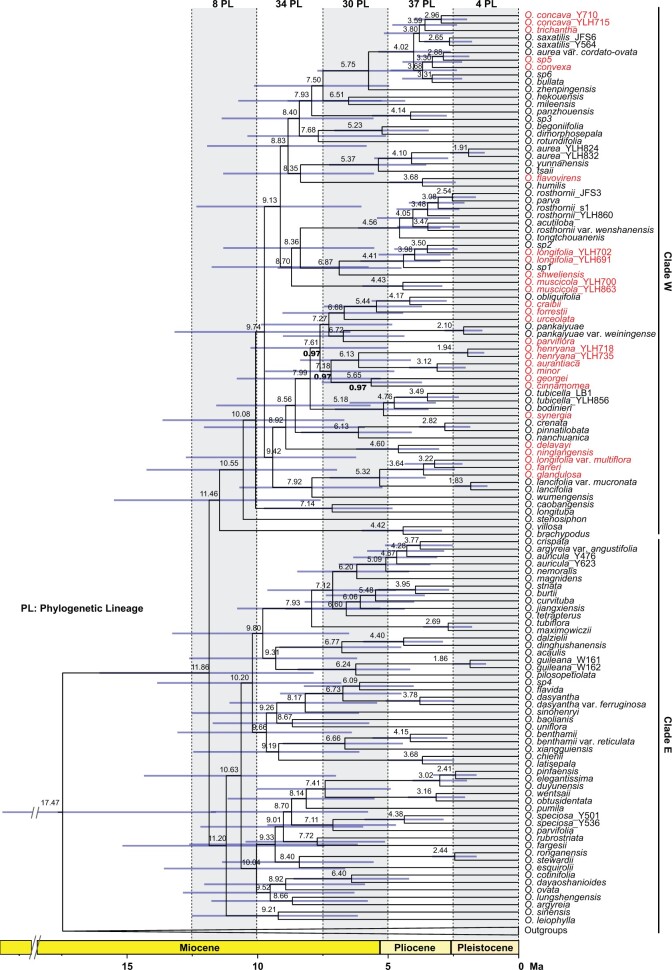
Bayesian time-calibrated phylogeny of *Oreocharis*. All nodes have maximal support values, except for three nodes with PP $=$ 0.97 (values shown in bold). Node labels indicate median age estimates. The species distributed in the HDM are shown in red. At the top of the chronogram, the number of speciation events (PL $=$ phylogenetic lineages) per 2.5 million year time slice is provided.

Bayesian analyses of divergence times performed with BEAST reached convergence as the ESS for the each parameter was greater than 200. The inferred phylogeny with BEAST was supported with maximal node support, except for three nodes with PP $=$ 0.97 that were located within the subclade including many species from the HDM (Fig. 4). The divergence time estimates yielded a crown age for *Oreocharis* of $\sim$11.86 Ma (95% HPD: 7.86–16.06 Ma), which corresponds to the middle to the late Miocene. This age estimate was very similar to that obtained in a previous study (Roalson and Roberts 2016). The divergence times for Clade W and Clade E were very close: $\sim$11.46 Ma (95% HPD: 7.59–15.51 Ma) and $\sim$11.20 Ma (95% HPD: 7.44–15.18 Ma), respectively. Overall, the LTT plot showed rapid species diversification early in the history of *Oreocharis*, followed by a slowdown of diversification towards the present (Supplementary Appendix S10 available on Dryad).

#### Diversification

**Figure 5.**
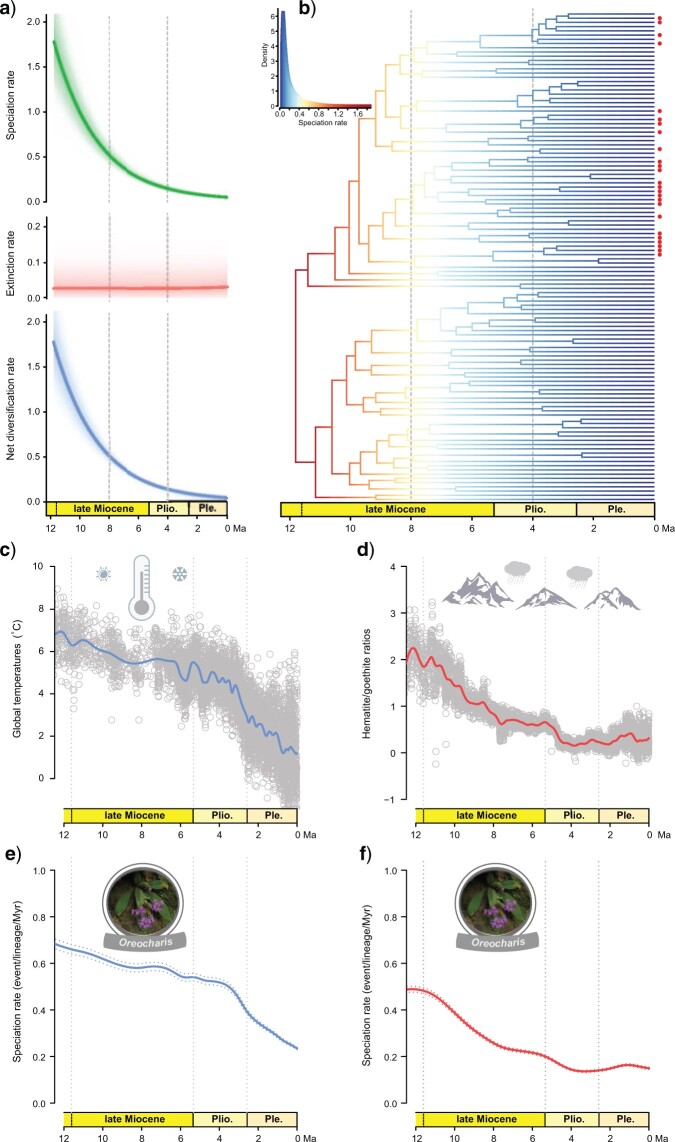
Diversification dynamic and macroevolutionary patterns of *Oreocharis*. a) Temporal variation in speciation, extinction, and net diversification rates inferred with BAMM. b) Maximum a posteriori probability shift configuration represented as a phylorate plot showing variation in speciation rates across the phylogeny of* Oreocharis*; blue represents low rates and red represents high rates. Each unique color section of a branch represents the mean of the marginal posterior density of speciation rates on a localized segment of a phylogenetic tree. The species distributed in HDM are noted with red dots. c, d) Past fluctuations of temperatures and East Asian monsoons since the late Miocene (data plotted from Zachos et al. 2008 and Clift et al. 2014, respectively). The hematite/goethite proxy, which is positively correlated to the strength of East Asian monsoons, is measured by 565 and 435 nm wavelengths in the color spectra of the freshly cut core from the ODP Site 1148 in the South China Sea (Clift et al. 2014). e, f) Speciation rates through time for *Oreocharis* obtained from the relationship between speciation rate and paleotemperatures e) and East Asian monsoons f) estimated using the approach of Condamine et al. (2013, 2019). The best model indicates strong positive correlations between speciation and past temperatures and East Asian monsoons, respectively. Plio. $=$ Pliocene; Ple. $=$ Pleistocene.

No significant rate shift was identified with BAMM, which showed a single macroevolutionary rate explaining the diversification of *Oreocharis* over time. The analyses indicate an early-burst of speciation with high initial speciation rates followed by a steady decline of speciation toward the present and a constant extinction rate since the late Miocene (Fig. 5). The *RPANDA* analyses supported a diversification model with time-variable rates that outperformed constant-rate models (Table 1). The BAMM and *RPANDA* analyses are congruent on inferring a decrease in speciation rate through time and a constant (but low) extinction rate.

**Table 1.** Results of RPANDA diversification analyses for *Oreocharis*.

**Model type**	**Model descriptions**	**Rate variation**	**NP**	**logL**	**AICc**	$\Delta$ **AICc**	**AIC** $\omega$	$\lambda_{0}$	$\alpha$	$\mu_{0}$	$\beta$
**Constant rate**	**Yule model**	**Constant**	**1**	$-355.729$	**713.490**	**48.822**	0	**0.1870**	**—**	**—**	**—**
	Constant BD	Constant	2	$-355.729$	715.554	50.886	0	0.1870	**—**	0	**—**
**Time dependence**	**B variable no D**	**Exponential**	**2**	$-337.617$	**679.330**	**14.662**	**2e** $-$ **04**	**0.0976**	**0.1371**	**—**	**—**
	B variable D constant	Exponential	3	$-337.618$	681.427	16.759	4e$-$04	0.0976	0.1371	0	**—**
	B constant D variable	Exponential	3	$-355.730$	717.651	52.983	0	0.1870	**—**	0	0.0534
	B variable D variable	Exponential	4	$-337.617$	683.557	18.889	1e$-$04	0.0976	0.1370	0	$-0.0184$
**Monsoon dependence**	**B variable no D**	**Exponential**	**2**	$-347.130$	**698.355**	**33.687**	0	**0.1340**	**0.6543**	**—**	**—**
	B variable D constant	Exponential	3	$-347.146$	700.484	35.816	0	0.1331	0.6662	0	**—**
	B constant D variable	Exponential	3	$-355.783$	717.757	53.089	0	0.1868	**—**	0	0.0479
	B variable D variable	Exponential	4	$-347.137$	702.597	37.929	0	0.1334	0.6607	0	0.0407
**Temperature dependence**	**B variable no D**	**Exponential**	**2**	$-330.286$	**664.668**	0	**0.679**	**0.0395**	**0.3569**	**—**	**—**
	B variable D constant	Exponential	3	$-330.287$	666.766	2.098	0.238	0.0395	0.3571	0	**—**
	B constant D variable	Exponential	3	$-355.782$	717.757	53.089	0	0.1868	**—**	0	0.0442
	B variable D variable	Exponential	4	$-330.287$	668.896	4.228	0.082	0.0395	0.3567	0	0.0218

*Notes:* The best fit models for each birth–death series that explain the phylogeny of the group are displayed in bold. Overall, the model with temperature-dependent speciation and no extinction is the best fitting model (AIC$\omega =$ 0.679). Values represent the mean of each parameter as estimated from 100 randomly selected trees of the posterior distribution of the dating analyses. *Abbreviation:* B $=$ birth or speciation; D $=$ death or extinction; NP $=$ number of parameters in the model; logL $=$ the log-likelihood of the model; AICc $=$ the corrected Akaike information criterion; $\Delta$AICc $=$ the difference in AICc between the model with the lowest AICc and the others; AIC$\omega$  $=$ Akaike weight; $\lambda_{0} =$ speciation rate at present; $\alpha =$ shape of speciation rate; $\mu_{0} =$ extinction rate at present; $\beta =$ shape of extinction rate.

The CoMET model in *TESS* also showed a slowdown of speciation rate with a constant extinction rate and no sudden extinction events (Supplementary Appendix S11 available on Dryad). The speciation rate estimated with CoMET was similar to BAMM, although CoMET inferred two distinct phases of slowdown. This is likely because BAMM uses rate smoothing through time (autocorrelated rates) while CoMET estimates rate using a time-continuous mode (Condamine et al. 2018).

The diversity-dependent analyses revealed that the best model for diversification was the linear diversity-dependent model without extinction (AICc$_{DDL\, }=$ 532.30). This model indicates that diversity-dependent processes may impose limitations on the diversification of *Oreocharis*, which was estimated to have a carrying capacity of 129 species (Table 2), suggesting that the genus may be reaching its ecological limits of diversification.

**Table 2.** Results of the diversity-dependent diversification (DDD) analyses for *Oreocharis*.

**Model**	**NP**	$-$ **logL**	**AICc**	$\Delta$ AICc	$\lambda$	$\mu$	**K**
**DDL**	**2**	**264.0976**	**532.301**	**0**	**0.6862**	**—**	**128.86**
DDL$+$E	3	264.0976	534.409	2.108	0.6861	0	128.86
DDX$+$E	3	320.3490	646.912	112.503	0.7018	0.1058	153.51
DD$+$EL	3	315.6576	637.529	103.120	0.2035	0.0254	20076.49
DD$+$EX	3	333.0496	672.313	137.904	0.3176	0.1293	578.57

*Notes*: The model with the highest support (lowest AICc) is shown in bold.

*Abbreviation:* $\lambda =$ speciation rate; µ_0_ = extinction rate; K $=$ estimated carrying capacity (i.e., ecological limits).

We compared the fit of four diversification models (i.e., constant-rate, time-dependent, monsoon-dependent, and temperature-dependent models) to determine which model better explains the diversification of *Oreocharis*. The paleoenvironment-dependent analyses showed that the speciation rate of *Oreocharis* positively correlates with both past temperatures ($\alpha =$ 0.3569; Fig. 5c,d) and East Asian monsoons ($\alpha =$ 0.6543; Fig. 5e,f). The most likely models were the temperature- and East Asian monsoons-dependent ones with two parameters (AICc $=$ 664.67 and 698.36, respectively). Comparing all models using AICc, we found that the best paleoenvironmental model was the one including the past temperature variation ($\Delta$AICc $=$ 33.69; Table 1). The other three models (constant-rate models, time-dependent models and the monsoon-dependent models) performed less well. Thus, the speciation rates of *Oreocharis* have decreased over time as the climate cooled toward the present (Fig. 5c,d), and BAMM, *RPANDA*, and CoMET models corroborated this slowdown in diversification. Smoothing the temperature curve decreased the fit for the temperature-dependent model ($\Delta$AICc $=$ 13.61; Table 1 and Supplementary Appendix S12 available on Dryad), showing that the support for the temperature-dependent model was likely not a false positive.

The BiSSE analyses suggested that the best-fitting model was a model in which speciation and transition rates were variable and extinction remained equal between the species distributed in HDM and non-HDM (AICc $=$ 709.27; the second best model with all variable parameters had an AICc $=$ 711.49; $\Delta$AICc $=$ 2.22; Table 3). Under the best BiSSE model, we found that the diversification rates were significantly different between the HDM and non-HDM regions with 0.32 versus 0.06 events/lineage/Ma, respectively (Table 3, Supplementary Appendix S13 available on Dryad). Moreover, the transition rate from HDM to non-HDM regions was 0.23 events/lineage/Ma but could not be detected in the opposite direction. Notably, we recovered a significant difference in diversification rates between the HDM and non-HDM regions regardless of the constraint applied on the root state (Table 3, Supplementary Appendix S14 available on Dryad). The HiSSE analyses revealed that the model including a hidden effect on diversification of state 1 (distributed in the HDM region) was strongly supported against the other SSE models (AICc $=$ 685.58 vs. AICc $=$ 698.22) for the second best estimated that the HDM species diversified about ten times faster than non-HDM species (0.65 vs. 0.065 events/lineage/Ma; Supplementary Appendix S15 available on Dryad), and also indicated that the diversification of HDM species was maybe influenced by other (unmeasured) traits, that is abiotic factors or intrinsic and/or extrinsic biotic factors. Randomization analyzes of the trait data set showed that the HiSSE-based results were robust to type-I error (Supplementary Appendix S16 available on Dryad), indicating that our inferences are unlikely to be biased.

**Table 3.** Results of the binary state speciation and extinction (BiSSE) analyses, while testing with different root states for *Oreocharis*.

**Root state**	**Model**	**NP**	**logL**	**AICc**	$\Delta$ **AIC**	$\lambda_{\mathbf{non-HDM}}$	$\lambda_{\mathbf{HDM}}$	µ_${\mathbf{non-HDM}}$_	µ_${\mathbf{HDM}}$_	**q** $_{\mathbf{non-HDM\to HDM}}$	**q** $_{\mathbf{HDM\to non-HDM}}$
null	Null model	3	$-370.836$	747.886	38.62	0.1722	—	0	—	0.0267	—
	lambda.free	4	$-370.692$	749.745	40.479	0.1766	0.1500	0	—	0.0269	—
	mu.free	4	$-370.836$	750.032	40.766	0.1723	—	0	0	0.0267	—
	q.free	4	$-359.517$	727.394	18.128	0.1722	—	0	—	0	0.1291
	lambda.mu.free	5	$-370.692$	751.93	42.664	0.1765	0.1500	0	0	0	—
	**lambda.q.free**	**5**	$-349.36$	**709.266**	**0**	**0.0579**	**0.3171**	**0**	—	**0**	**0.2344**
	mu.q.free	5	$-359.525$	729.595	20.329	0.1722	—	0	0	0	0.1292
	all.free	6	$-349.36$	711.491	2.225	0.0578	0.3171	0	0	0	0.2344
0 ($=$non-HDM)	Null model	3	$-370.836$	747.886	21.489	—	0.1722	—	0	—	0.0267
	lambda.free	4	$-370.692$	749.744	23.347	0.1766	0.1500	—	0	—	0.0269
	mu.free	4	$-370.836$	750.032	23.635	—	0.1723	0	0	—	0.0267
	q.free	4	$-362.329$	733.019	6.622	—	0.1722	—	0	0.0037	0.1200
	lambda.mu.free	5	$-370.692$	751.93	25.533	0.1766	0.1500	0	0	—	0
	**lambda.q.free**	**5**	$-357.926$	**726.397**	**0**	**0.1400**	**0.2515**	—	**0**	**0.0035**	**0.1257**
	mu.q.free	5	$-362.331$	735.207	8.810	—	0.1722	0	0	0.0038	0.1202
	all.free	6	$-357.926$	728.622	2.225	0.140	0.2515	0	0	0.0035	0.1257
1 ($=$HDM)	Null model	3	$-378.746$	763.706	54.437	—	0.1721	—	0	—	0.0329
	lambda.free	4	$-378.627$	765.614	56.345	0.1767	0.1509	—	0	—	0.0329
	mu.free	4	$-378.746$	765.852	56.583	—	0.1722	0	0	—	0.0329
	q.free	4	$-359.518$	727.397	18.128	—	0.1722	—	0	0	0.1291
	lambda.mu.free	5	$-378.627$	767.799	58.530	0.1767	0.1509	0	0	—	0
	**lambda.q.free**	**5**	$-349.362$	**709.269**	**0**	**0.0578**	**0.3174**	—	**0**	**0**	**0.2356**
	mu.q.free	5	$-359.523$	729.591	20.322	—	0.1722	0	0	0	0.1292
	all.free	6	$-349.36$	711.491	2.222	0.0578	0.3171	0	0	0	0.2344

*Notes:* The model with the highest support (lowest AICc) is shown in bold.

*Abbreviation:* q, transition rate.

### Ancestral Areas of Origin

**Figure 6.**
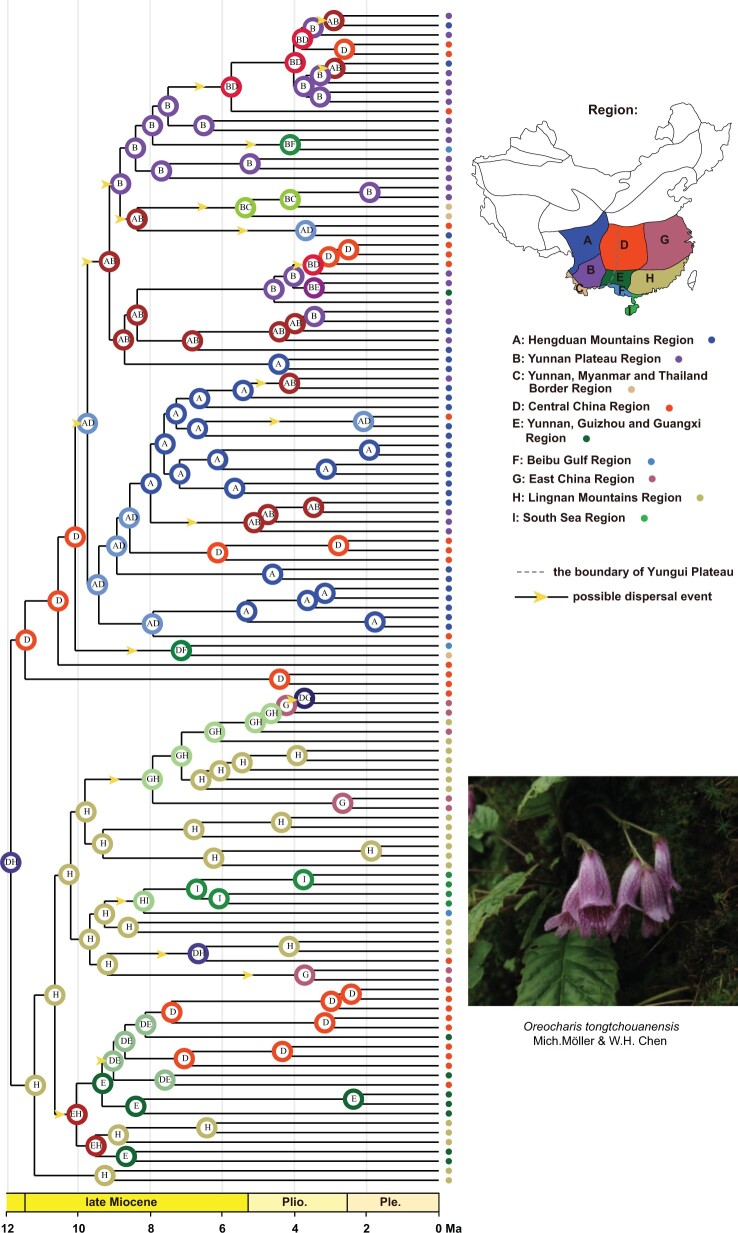
Estimation of the biogeographic history of *Oreocharis*. The map in the top right corner represents the nine floristic divisions (a–h) according to Huang et al. (2016) where *Oreocharis* was sampled. The dotted grey line indices the boundary of Yungui Plateau. The yellow arrows indicate the possible dispersal events. Pictured in the right corner is *Oreocharis tongtchouanensis* occurring in the Hengduan Mountains (© Lihua Yang).

The DEC-based biogeographic analysis revealed Central China and the Lingnan Mountains Region as the most likely ancestral area of origin of *Oreocharis* with maximal probability (Fig. 6; see Supplementary Appendix S17 available on Dryad for probability at each node). The Clade W most likely originated in Central China region, while the Clade E originated in the region of Lingnan Mountains, indicating a vicariant event early in the history of *Oreocharis*. The Clade W colonized the Hengduan Mountains $\sim$10 Ma ago from Central China. At least six dispersal events with high probability (p >0.70) are detected from Hengduan Mountains to other regions, but no other dispersal events into the Hengduan Mountains are detected from other regions, suggesting rare colonizations events, which is similar to the transition rate (q$_{non-HDM\to HDM})$ estimated with the BiSSE analyses (Table 3). Therefore, there is an incompatibility between the root ancestor as inferred with DEC (non-HDM region) and the inference of zero dispersal event out of non-HDM region as estimated with the SSE models. We also counted six vicariance events and seven dispersal events in the Clade E, and 16 vicariance events and 15 dispersal events in the Clade W (Fig. 6). A more detailed biogeographic history is available on Dryad (Supplementary Appendix S17 available on Dryad).

## Discussion

Reconstructing the phylogeny of a lineage that has undergone a rapid radiation and analyzing the factors potentially involved in its diversification represent challenging goals for current evolutionary biology but are critical for understanding patterns of diversity in systems, such as mountains, that often comprise many clades diversifying rapidly (e.g., Drummond et al. 2012; Favre et al. 2015; Lagomarsino et al. 2016; Kong et al. 2017; Esquerré et al. 2019; Muellner-Riehl 2019). In the last decade, the proliferation of new sequencing technologies has allowed producing hundreds or even more loci for inferring robustly resolved phylogenetic hypotheses across the tree of life, thus, improving our understanding of biological evolution (McCormack et al. 2013; Bragg et al. 2018; Roycroft et al. 2020). Therefore, we employed transcriptomic data to attempt to disentangle the different historical processes and quantify the relative contribution of factors likely responsible for generating of the species-rich, primarily montane genus, *Oreocharis*, as an example of the analytical power of these modern approaches.

### Rapid Evolutionary Radiation of **Oreocharis**

Our results from BAMM, *RPANDA*, and CoMET revealed that *Oreocharis* experienced an early rapid radiation. The estimate of net diversification rates between 11.8 and 8 Ma is 1.03 species per million years (species/Myr). This rate is higher than in many documented examples of fast plant radiations in Southwest China, including of alpine bamboos (0.75 species/Myr; Poaceae; Ye et al. 2019), tribe Anastaticeae (0.17 species/Myr; Brassicaceae; Qian et al. 2018), *Hirculoideae* and *Kabschia* (0.455 and 0.471 species/Myr; Saxifragaceae; Ebersbach et al. 2017), and 17 angiosperm clades (ca. 0.12 species/Myr; Xing and Ree 2017); or in other biodiversity hotpots; for example, *Fosterella* within the Andean Mountains (0.40 species/Myr; Bromeliaceae; Wagner et al. 2013). However, it is slower than rates detected in Andean *Lupinus* (2.49-3.72 species/Myr; Fabaceae; Hughes and Eastwood 2006) and Andean bellflowers (1.83 species/Myr; Campanulaceae; Lagomarsino et al. 2016).

Early bursts followed by slowdowns in diversification have been hypothesized as the main feature of adaptive radiations (Gavrilets and Losos 2009; Glor 2010; Yoder et al. 2010; Moen and Morlon 2014), because early bursts of diversification may be driven by the exploitation of new ecological niches, therefore allowing lineages to enter new adaptive zones until the newly available niche space reaches saturation (Yoder et al. 2010). The early burst of diversification followed by a steady decline of diversification, without a corresponding increase in extinction rate, detected in *Oreocharis* seemingly agrees with this hypothesis.

However, some other biological explanations might also underlie the slowdown of diversification, such as the geographic context of diversification and environment-driven bursts of speciation (Moen and Morlon 2014). Alternatively, adaptive processes might not drive the rapid early diversification of *Oreocharis* and, instead, repeated allopatric speciation could have occurred through vicariant geographic processes followed by range shifts without adaptive divergence. The biogeographic analyses support the hypothesis of an evolutionary history impacted by vicariance. Interestingly, we estimated a similar number of allopatric speciation and speciation by range expansion.

Irrespective of the manner in which *Oreocharis* has radiated, the decrease of diversification rate in the group implies that ecological niche space is limiting and is a likely a cause of the signal of diversity dependence recovered with the *DDD* model. Indeed, we estimated an extant carrying capacity close to the extant number of species (Table 2), which lends support to the hypothesis that the diversification slowdown in *Oreocharis* since the late Miocene may have resulted from the rapid filling of available ecological niches.

The diversification dynamic of *Oreocharis* resembles the pattern observed in the closely related genus, *Primulina*, in which repeated geographic isolation within karst landscapes resulted in nonadaptive diversification with a conserved morphology among species (Kong et al. 2017). Additionally, the diversification pattern of both genera is consistent with the scenario of environment-driven speciation, in which rapid climate change can induce pulses of high speciation rates across the regional species pool and decreases after the change of environmental conditions (Moen and Morlon 2014; Kong et al. 2017).

### Environment-driven Speciation: The Impact of Mountain Building and Climate Change

Previous studies have proposed mountain building as a predominant driver in triggering biological evolution (Hoorn et al. 2013; Antonelli et al. 2018), especially for the diversification of the HDM flora (Sun et al. 2017 and references therein). Only a few recent studies have explicitly modeled the diversification dynamics in mountains over time (Favre et al. 2016; Ebersbach et al. 2017; Xing and Ree 2017; Condamine et al. 2018; Ye et al. 2019; Ding et al. 2020). One limitation of these studies is the lack of comparisons of diversification rates between different mountain lineages (but see Xing and Ree 2017; Ding et al. 2020).

In this study, we recovered that the genus originated in the mountains of Central China and the Lingnan Mountains with two main lineages separated by the Yungui Plateau (Fig. 6, Supplementary Appendix S1 available on Dryad), which is an upland boarding the HDM to the east. The DEC-based analyses estimated an allopatric speciation with the two sister lineages inheriting each half of the ancestral range (Clade W in Central China and Clade E in Lingnan Mountains). This suggests that an early vicariance, likely related to mountain building, climate, or a combination of the two, was a driver of the initial lineage divergence within *Oreocharis*. Central, southern and south-eastern China have been characterized by relative tectonic stability since the late Cenozoic (Hsü 1983; López-Pujol et al. 2011). If mountain building did directly trigger the initial diversification, we would expect different diversification rates between the western lineage and the eastern lineage due to the different mountain building history. However, no shift in diversification rates was detected across the genus (although HDM lineages have different diversification rates as discussed below). Hence, we argue that the HDM uplift was unlikely the direct and major driver of the rapid diversification and accumulation of species diversity observed in *Oreocharis*. This hypothesis differs from those in recent studies of other plant lineages that showed accelerated diversification rates coinciding with the uplift of HDM (Favre et al. 2016; Ebersbach et al. 2017; Xing and Ree 2017; Ye et al. 2019), suggesting that the effect of mountain building on rapid diversification of the HDM flora is probably taxon dependent. Unlike previous studies, which focused on plant groups from the high-altitude habitats (alpine/subalpine zone), most *Oreocharis* species occupy subtropical, humid, and monsoonal forests at low to medium elevations (1,000–2,500 m), and their diversification might have taken place after the formation of mountain systems. Indeed, 61% (11/18) of the speciation events in the HDM occurred more recently than 5 Ma (Fig. 4), and thus postdate the main uplift phase of the HDM that reached its current elevation by the early Oligocene (Su et al. 2018; Spicer et al. 2020, 2021; Xiong et al. 2020). Given the likely mountainous origin, it is also plausible the ancestor of *Oreocharis* was already adapted to a mountain habit, and thus colonizing the HDM did not provide an ecological opportunity followed by a diversification upshift, but instead the HDM allowed a more continuous speciation rate. Such a preadaptation pattern has been shown in some Neotropical orchids colonizing and diversifying in the Andes (Pérez-Escobar et al. 2017).

We do not rule out a contributing role of mountain building in promoting diversification of this group. The HDM uplift, subsequently deeply dissected by river incision (Nie et al. 2018), has created strong environmental heterogeneity, which promoted geographic isolation of populations and provided opportunities for allopatric speciation, then stimulated the higher *in situ* diversification rate than in other regions (Xing and Ree 2017; Spicer et al. 2020, 2021). Furthermore, the increased topographic complexity may have induced local climate changes and consequently promoted speciation via local adaptation. For example, the BiSSE and HiSSE analyses indicated that the HDM lineages diversified significantly faster than the non-HDM lineages. Importantly, the higher diversification rates in the HDM has no notable effect on the overall diversification rate across the whole genus, that is no shift of diversification rates was detected due to the uplift of the HDM (Fig. 5b).

The DEC inference (ancestor being in non-HDM) and the SSE estimate (dispersal rate out of non-HDM region $=$ 0) seem incompatible as it is unlikely for a clade to originate in one region, colonize adjacent regions, but show null dispersal rate out of this center of origin. We suspect that these opposite result may be explained by the difference in character states analyzed in both analyses (2 states in SSE models vs. 9 states in DEC model). It is important to note that DEC inferred a few dispersal events from non-HDM regions to the regions including HDM (e.g., D to AD about 10 Ma; Fig. 6), which can explain the low dispersal rate out of non-HDM region, but certainly not zero. It is also possible that the SSE models failed to recover a nonzero dispersal rate because such events were rare; an issue that can stem from the data (too few colonization events) not only from the model. Finally, clades including HDM species likely originated by vicariance (six times, Fig. 6; Supplementary Appendix S17 available on Dryad), and the BiSSE and HiSSE models may be inappropriate in this situation.

The onset and intensification of Asian monsoons since the Miocene has been suggested to have impacted species diversification of many plant lineages within East Asia (Wang et al. 2012; Yao et al. 2016; Kong et al. 2017; Lin et al. 2019; Ding et al. 2020; Li et al. 2021), but there have been few attempts to directly test for a relationship between diversification rate and Asian monsoons (but see Kong et al. 2017). Using a paleoenvironment-dependent diversification model, we found that the diversification rate within *Oreocharis* is highly correlated with the strength of the East Asian monsoons. Specifically, the origin and early burst of diversification was estimated around the beginning of the late Miocene (11.86 Ma; Fig. 4), a period coinciding with the first strengthening event of the East Asian monsoon around 13 Ma (Sun and Wang 2005; Wan et al. 2007). Previous studies have suggested that the Asian monsoons have affected the amount and seasonality of precipitation in East Asia (Quan et al. 2011; Liu and Dong 2013; Farnsworth et al. 2019; Li et al. 2021) and provided ideal conditions for the development of new ecological niches for mountain plants. We infer that the novel ecological niches created by the establishment of the East Asian monsoons may have first triggered the radiation of *Oreocharis* (Fig. 5f), which diversified quickly into new suitable habitats created as the East Asian monsoons first strengthened across the region. When speciation rates are the highest, the biogeographic analyses inferred nine dispersal events (out of 22 in total) between 11.8 and 8 Ma (2.4 events/Myr; Fig. 6). However, the rate of diversification probably declined due to reduced opportunities when the monsoon entered its weakened stage, which is accompanied by a decrease of dispersal events (13 between 8 Ma and the present; 1.6 events/Myr).

Despite different hypotheses on the formation and evolution of the East Asian monsoons (Lu et al. 2010; Molnar et al. 2010; Liu and Dong 2013), global cooling might provide the best explanation for the formation of the monsoon in East Asia during the Cenozoic (Lu and Guo 2014; Miao et al. 2012, 2016; Zhang et al. 2018b). Therefore, it is not surprising the diversification rates of *Oreocharis* were positively associated with both global cooling and the East Asian monsoons. Additionally, the species of *Oreocharis* favor humid habitat. Both of those suggest that *Oreocharis* thrived during a warm and wet period as it was in the mid and beginning of the late Miocene. Our results reveal a positive dependence of speciation to past temperatures of *Oreocharis*, indicating that speciation rates tend to be higher under warm climates (Condamine et al. 2019). There is evidence for a link between warm climate and species diversity in plants (e.g., Jaramillo et al. 2006). Mechanistically, such an evolutionary relationship would imply that higher temperature could lead to higher mutation rates, which could lead to within-species divergence and eventually speciation (Allen et al. 2006). A similar pattern of diversification dynamic associated with paleoenvironmental changes were also reported in the temperate bamboos (Guo et al. 2021) and in *Primulina*, a genus closely related to *Oreocharis* that also widely distributed in subtropical China (Kong et al. 2017). Additionally, the high divergent floral characters (Fig. 1a) suggest that other drivers, such as pollination syndrome, may also have an important role in the speciation process of *Oreocharis* (Möller et al. 2011), which has been shown in Gesneriaceae (Roalson and Roberts 2016).

### ILS is Common Throughout the Rapid Radiation of **Oreocharis**

A robust and well-resolved phylogenetic framework is a cornerstone for the study of macroevolutionary patterns and must be derived from extensive geographic and taxonomic sampling to provide high confidence in tree topologies and species relationships as well as in branch length estimations. Unfortunately, disentangling phylogenetic relationships arising during rapid radiations remains a significant challenge due to the confounding effects of ILS and hybridization (Maddison and Knowles 2006; Degnan and Rosenberg 2006, 2009; Knowles and Chan 2008; Giarla and Esselstyn 2015; Alexander et al. 2017; Mclean et al. 2019; Roycroft et al. 2020). Previous attempts to infer phylogenetic relationships in *Oreocharis* based on ITS and chloroplast DNA sequences resulted in very poor resolution and low statistical support for shallow nodes (Möller et al. 2011; Chen et al. 2014). Furthermore, with such a small number of loci it yields very little statistical power and is inadequate to deal with inconsistencies related to ILS and gene flow.

This study provides the most comprehensive phylogeny of *Oreocharis* to date based on a transcriptomic data set. Our analyses indicated several well-supported and relatively consistent subclades, and both concatenation and coalescent-based approaches generated a species tree with high statistical support for nodes. Nonetheless, our results show widespread conflict between individual gene trees and the species tree, especially for the placements of short branches (Supplementary Appendices S3b and S8 available on Dryad). Based on our large genomic data set, we established that this conflict can be largely explained by the presence of extensive genome-wide ILS in *Oreocharis*. Our results provide clear evidence that substantial ILS, accompanying this rapid radiation, has shaped and complicated the evolutionary history of this group. This result deserves further studies, potentially using other data, such as from maternally inherited chloroplast genomes.

## Conclusions

Here, we present a taxonomically comprehensive dated phylogeny of *Oreocharis* and show evidence for early rapid radiation and extensive ILS during the evolutionary history of the genus. This group experienced a burst of speciation in its early evolutionary history, followed by a decline in diversification rate toward the present. We found that the warm and humid climate of the mid-Miocene, together with the East Asian monsoons and global temperature change may have functioned synchronously as the primary drivers of diversification in *Oreocharis*, although mountain building may have indirectly affected species diversification in the HDM. Our study highlights the importance of past climatic changes, combined with mountain building, in creating strong environmental heterogeneity and driving plant diversification, and suggests that the biodiversity within the HDM cannot directly be attributed to mountain uplift. Future studies may investigate whether the diversification patterns identified here are mirrored by patterns in other plant lineages and/or in other mountain systems.

## Supplementary Material

Data available from the Dryad Digital Repository: https://doi.org/10.5061/dryad.9cnp5hqjf.
